# Therapy-Related Myeloid Neoplasms in Patients Treated for Hodgkin Lymphoma

**DOI:** 10.4084/MJHID.2011.046

**Published:** 2011-10-24

**Authors:** D.A. Eichenauer, A. Engert

**Affiliations:** 1 First Department of Internal Medicine, University Hospital of Cologne, Cologne, Germany; 2 German Hodgkin Study Group (GHSG), Cologne, Germany

## Abstract

Hodgkin lymphoma (HL) is a malignancy of the lymphatic system with an incidence of 2-3/100.000/year in developed countries. With modern multi-agent chemotherapy protocols optionally combined with radiotherapy (RT), 80% to 90% of HL patients achieve long-term remission and can be considered cured. However, current standard approaches bear a considerable risk for the development of treatment-related late effects. Thus, one major focus of current clinical research in HL is reducing the incidence of these late effects that include heart failure, infertility, chronic fatigue and therapy-related myelodysplastic syndrome/acute myeloid leukemia (t-MDS/t-AML). In previous analyses, t-MDS/t-AML after treatment for HL was associated with a poor prognosis. Nearly all patients died rapidly after diagnosis. However, more recent analyses indicated an improved outcome among patients with t-MDS/t-AML who are eligible for modern anti-leukemic treatment and allogeneic stem cell transplantation (aSCT). This article gives an overview of recent reports on the incidence and the treatment of t-MDS/t-AML after HL therapy and describes the efforts currently made to reduce the risk to develop this severe late effect.

## Introduction

**Figure 1:**
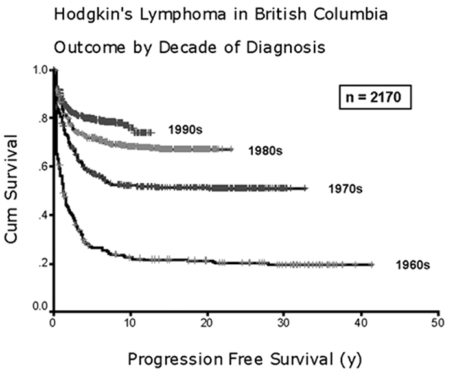
Progression-free survival among Hodgkin lymphoma patients treated in British Columbia during the indicated decades (adopted from Connors, Hematology Am Soc Hematol Educ Program, 2003)

Hodgkin lymphoma (HL) is a malignancy of the lymphatic system with an incidence of 2-3/100.000/year in Europe and North America.^1^ The disease generally occurs in all age groups but young adults are most often affected.^2^ As a result of substantial treatment improvements in the past decades including the introduction of highly effective multi-agent chemotherapy protocols and the optimization of radiation fields and doses, HL has become one of the best curable adult malignancies. Irrespective of the initial stage, 80% to 90% of patients achieve long-term remission (Figure 1).^3^–^5^ This has led to a steadily growing number of HL survivors. Since these survivors often suffer from treatment-related late effects such as heart failure, infertility, chronic fatigue and secondary malignancies, reducing the frequency of long-term sequelae without compromising treatment efficacy has become one of the major challenges of current clinical research in HL.^6^–^9^ Therapy-related myelodysplastic syndrome/acute myeloid leukemia (t-MDS/t-AML) represents one of the most severe late effects after HL treatment and has been associated with a particularly poor prognosis. Nearly all patients died rapidly after diagnosis.^10^

However, incidence and prognosis of t-MDS/t-AML after HL treatment may change in the coming years. Response-adapted treatment strategies that are currently being evaluated in clinical trials will potentially lead to a decrease of cumulative chemotherapy and radiation doses in many HL patients. Thus, the risk to develop t-MDS/t-AML will be reduced. Improvements in the treatment of t-MDS/t-AML mainly due to the increased availability and the more efficient use of allogeneic stem cell transplantation (aSCT) will probably have a positive impact on the prognosis of the mostly young patients diagnosed with t-MDS/t-AML after HL treatment.

This review aims at giving an overview of relevant analyses on the development, incidence, clinical course and treatment of t-MDS/t-AML after HL. Current strategies to reduce the risk of t-MDS/t-AML are also discussed.

## Leukemogenic drugs in HL treatment

ABVD (adriamycin, bleomycin, vinblastine, dacarbazine) and BEACOPP (bleomycin, etoposide, adriamycin, cyclophosphamide, vincristine, procarbazine, prednisone) are the chemotherapy protocols most widely used for the first-line treatment of adult patients with HL.^11^–^12^ Both regimens include alkylating agents, namely dacarbazine in the ABVD protocol and cyclophosphamide and procarbazine in the BEACOPP schema. Alkylating agents are known to be leukemogenic. Secondary leukemias induced by this drug class mostly occur three to eight years after exposition and are often preceded by a pre-leukemic phase that is characterized by myelodysplasia. Chromosome aberrations are common in t-MDS/t-AML induced by alkylating agents. Losses of chromosome 5 or chromosome 7 as well as deletions of the long arm of the same chromosomes are most frequently observed.^13^

Topoisomerase-II-inhibitors represent another drug class with leukemogenic potential used in the treatment of HL. Particularly etoposide is frequently applied. Besides first-line protocols such as BEACOPP it is also contained in high-dose regimens used in the salvage setting, BEAM (BCNU, etoposide, ara-c, melphalan) for instance.^12^,^14^ In comparison with alkylating agents, secondary leukemias induced by topoisomerase-II-inhibitors are characterized by a more rapid development after exposure. Thus, the median latency period between treatment with topoisomerase-II-inhibitors and diagnosis of secondary leukemia is about two years; a pre-leukemic phase with myelodysplastic alterations in the bone marrow is usually not observed. However, similar to alkylating agents, chromosomal aberrations are also often found in secondary leukemias after treatment with topoisomerase-II-inhibitors. Translocations at the myeloid-lymphoid leukemia (MLL) gene locus 11q23 represent the most frequent aberration.^13^

## Incidence, clinical course and treatment of t-MDS/t-AML in HL patients

**Figure 2:**
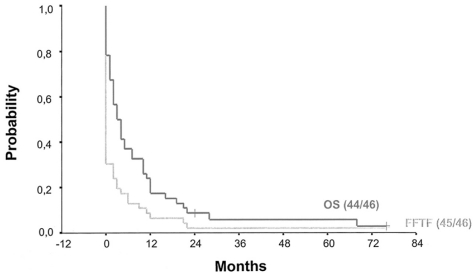
Freedom from treatment failure and overall survival among patients with t-MDS/t-AML treated for Hodgkin lymphoma (adopted from Josting et al., J Clin Oncol, 2003)

The two most recent reports on t-MDS/t-AML in HL patients come from the German Hodgkin Study Group (GHSG). Josting and colleagues screened 5.411 patients treated within GHSG clinical trial protocols between 1981 and 1998 for the development of t-MDS/t-AML. Results were published in 2003. At a median follow-up of 55 months, 46 patients had developed t-MDS/t-AML; the cumulative risk to develop t-MDS/t-AML was 1%. In the majority of cases, HL treatment had consisted of ABVD or ABVD-based chemotherapy protocols mostly combined with consolidating radiotherapy (RT); some patients had received BEACOPP. The median time interval between HL treatment and the diagnosis of t-MDS/t-AML was 12.5 months. An evaluation of cytogenetic changes was performed in 15 patients. All of them had chromosomal abnormalities. Aberrations affecting chromosome 5 or chromosome 7 and the presence of an MLL-rearrangement were most often observed. Clinical outcome of patients with t-MDS/t-AML included in this analysis was poor with a median overall survival (OS) of only four months for the whole patient group and ten months for the nine patients who underwent aSCT. At 24 months, freedom from treatment failure and OS rates were 2% and 8%, respectively (Figure 2).^10^

An update analysis including patients treated according to GHSG trial protocols between 1993 and 2009 was presented in abstract form in 2010. In contrast to the report from Josting and colleagues, an increased portion of patients included in this analysis had received the intensive BEACOPP_escalated_ protocol representing the current standard of care for younger patients with advanced HL within the GHSG. A total of 11891 patients were screened for the occurrence of secondary myeloid neoplasms. Therapy-related MDS/AML had been diagnosed in 99 of them. However, 13 patients were excluded from final analysis due to a concurrent event prior to the diagnosis of t-MDS/t-AML so that 86 patients were eventually taken into account. Since the intensity of HL treatment appears to have a significant impact on the risk to develop t-MDS/t-AML, patients were divided into three subgroups. Patients from the first group had received no BEACOPP_escalated_ - containing chemotherapy, patients from the second group had received less than four cycles of BEACOPP_escalated_ and patients from the third group had received four of more cycles of BEACOPP_escalated_. As a result, the risk for the development of t-MDS/t-AML was significantly increased for patients treated with four or more cycles of escalated BEACOPP while the risk for patients from the other groups was comparable (1.5% vs 0.5% vs 0.3%). In comparison with the report from Josting and co-workers, a higher portion of patients included in the update analysis had received aSCT for the treatment of t-MDS/t-AML. These mostly young patients who had been eligible for intensive induction and/or conditioning protocols prior to aSCT showed an improved outcome. However, overall outcome after diagnosis of t-MDS/t-AML was still poor with a median survival of 7.2 months for the whole patient group.^15^

The improved treatment results reported for selected patients eligible for aSCT are in line with a recent analysis from Kayser and colleagues. The outcome of 200 patients with t-AML who were previously treated for different solid and hematologic malignancies was compared with the treatment results of 2653 patients with *de novo* AML. Relapse-free survival and OS were inferior in patients with t-AML with 4-year rates of 24.5% and 25.5%, respectively, compared to 37.9% and 39.5%, respectively, for patients with *de novo* AML. However, 40 of the 200 patients with t-AML included in the analysis received aSCT in first complete remission and had a 4-year OS rate of 42.6%. This is still inferior when compared with patients with *de novo* AML undergoing aSCT who had a 4-year OS rate of 58.0% but significantly better than reported in older analyses on t-AML.^16^

A similar analysis was performed by Litzow and coworkers. The outcome of 545 patients with t-AML and 323 patients with t-MDS who underwent allogeneic bone marrow or stem cell transplantation between 1990 and 2004 was analyzed. Disease-free survival and OS rates were 32% and 37%, respectively, at one year and 21% and 22%, respectively, at four years. At first sight, these results appear inferior in comparison with the data reported by Kayser and colleagues. However, the analyses are difficult to compare since Litzow and coworkers also included patients who received an allograft in the early 1990’s. Results achieved with aSCT at that time are not comparable with those observed today.^17^

In conclusion, the prognosis of patients with t-MDS/t-AML has apparently improved within the past years. The main reason for this improvement consists in the increased availability and the optimized use of aSCT.

## Prevention of late effects including t-MDS/t-AML

With current standard approaches consisting of multi-agent chemotherapy optionally combined with RT, patients diagnosed with HL achieve long-term remission and can be considered cured in more than 80% of cases.^3^–^5^ Since remission rates can hardly be improved, the reduction of acute and long-term side effects including secondary hematologic malignancies such as t-MDS/t-AML has become increasingly important in recent years.

Ongoing clinical trials aim at reducing the risk to develop late sequelae including t-MDS/t-AML without compromising treatment efficacy. Thus, response-adapted treatment strategies are being evaluated. Positron emission tomography (PET) is currently considered the most promising tool to distinguish between patients who are sufficiently treated with less aggressive approaches and patients who require standard or even intensified protocols.

Within the GHSG HD15 trial, patients with advanced HL were initially randomized to receive either eight cycles of BEACOPP_escalated_, six cycles of BEACOPP_escalated_ or eight cycles of BEACOPP-14. Then, a PET scan was performed in patients with residual lymphoma larger than 2.5 cm. Localized RT was confined to those patients with PET-positive residual disease. As a result, the negative predictive value of PET defined as the proportion of PET-negative patients without progression, relapse or RT within 12 months was 94.6%. Thus, it appears possible to restrict consolidating RT to patients with larger PET-positive residual lymphoma after intensive chemotherapy with escalated BEACOPP.^18^

In the ongoing GHSG follow-up trial, HD18 (NCT0051554), all patients receive two cycles of escalated BEACOPP before an interim PET is conducted. Then, patients with a negative PET are randomized between the standard treatment consisting of six further cycles of BEACOPP_escalated_ and a reduced treatment consisting of only two further cycles of BEACOPP_escalated_ while patients with insufficient metabolic response are randomized between the standard treatment and an intensified protocol consisting of six further cycles of escalated BEACOPP supplemented by the anti-CD20 antibody rituximab in the last five cycles.

In a trial conducted by an Italian Group (NCT00795613), treatment for patients with advanced HL is also stratified according to early interim PET. All patients initially receive two cycles of ABVD. Then, a PET scan is performed. Patients without detection of active disease continue treatment with ABVD while patients with PET-positive residual lymphoma switch to the more intensive BEACOPP_escalated_ protocol for the rest of treatment.

In addition to the trials mentioned, further studies investigating response-adapted strategies with the aim to reduce treatment intensity in patients with good initial response on the one hand and intensify treatment in high-risk patients on the other hand are currently recruiting patients.

Another possible way to reduce the incidence of t-MDS/t-AML may consist in choosing the HL treatment according to the patient’s predisposition to develop therapy-related secondary malignancies. However, no susceptibility factors such as certain single nucleotide polymorphisms (SNP) predicting the risk of the individual patient to develop t-MDS/t-AML have been identified to date. Analyses of larger patient series addressing this issue appear necessary but are pending.

## Summary

Within the past decades, HL has turned from an incurable disease to one of the adult malignancies with best cure rates. Thus, treatment efficacy can hardly be improved and the prevention of acute and long-term toxicity including lung and heart failure, temporary or permanent infertility and secondary malignancies including t-MDS/t-AML has become one of the major challenges.

Currently ongoing trials aim at reducing the cumulative treatment toxicity by using response-adapted strategies. Low-risk and high-risk patients are being distinguished according to the result of an interim PET. Once valid results from these trials will be available, treatment stratification based on early interim PET might become standard of care in HL therapy. Thus, cumulative chemotherapy and radiation doses could decrease in a relevant portion of patients.

Patients who are diagnosed with t-MDS/t-AML after HL treatment still have a poor prognosis although substantial therapeutic improvements were made in the past decade. These improvements are mainly due to the increased availability and the optimized use of aSCT. About 20% to 40% of patients with t-MDS/t-AML achieve long-term remission when treated with aSCT.^16^–^17^

## Conclusion

The major goals in connection with t-MDS/t-AML after treatment for HL consist in 1) the establishment of risk-adapted treatment strategies for HL and 2) the further optimization of treatment for patients diagnosed with t-MDS/t-AML.
